# Round the Hospitals

**Published:** 1919-09-20

**Authors:** 


### ROUND THE HOSPITALS.

We understand that the Dowager Marchioness of Dufferin and Ava has arranged for the reorganisation of the United Kingdom Branch of the Dufferin Association. An executive committee has been appointed under the chairmanship of the Hon. Mrs. Edwin Montagu, and in future all appointments made to the Women's Medical Service in India from this country will be made by this committee, after having been duly advertised. The Hon. Secretary is Miss L. M. Brooks, India Office, Whitehall, S.W. 1, to whom all applications should be made.

Like other branches of the medical and nursing service, the Countess of Dufferin's Fund for supplying female medical aid to the women of India suffered acutely last year from a shortage of recruits. Many qualified women doctors were needed to take

**ROUND THE HOSPITALS**—(*continued*).

up appointments in military hospitals, but the hospitals promoted by the fund have kept up their high standard, and there is growing appreciation of the British female medical service. An amusing feature of the report is the account given of the competition among Indian school-girls for essays on "The Care of the Baby." Sixty-eight schools competed, and twenty-two prizes were awarded. Old and new theories jostled each other strangely. One child recommended mutton bones as a diet for infants of six months, and another prescribed coffee and tea with several different kinds of patent foods at a time if the baby showed signs of sickliness; while the use of flannel binders for the feet was earnestly recommended. Many papers showed, however, that the good seed is growing.

---

THERE will be widespread satisfaction in nursing circles at the announcement of the grants, amounting to nearly a million pounds, made by the Joint War Committee of the British Red Cross Society and the Order of St. John to the hospitals and county associations. The recognition accorded to the nursing associations is particularly grateful. In nearly every instance their operations are severely hampered for lack of funds, and the grants, which total from £1,000 to £3,000, according to population, will enable much needed extensions to take place in their programme of work. Their principal item of expenditure is for the training of nurses for district and village work and for midwifery, and the grants will forward the development of training on good lines and supply a real stimulus. The stipulations which attend the distribution of grants are admirable, and we are confident that the county nursing associations will be able to demonstrate that full value will be reaped from the money which has fallen to their share from the Red Cross.

---

THE Committee appointed under the National Council of Women last February to examine into the Economic position of nurses has now issued its report. It will be remembered that this Committee was appointed subsequently to the one formed under the College of Nursing to report on salaries and conditions of employment of nurses, and that an adroit attempt was made to capture the College Committee and supersede its functions by this other body. We never swerved from our opinion that the enquiry was a proper one for the College of Nursing to undertake, and that its supersession by a body nominated under the ægis of a society holding no mandate in nursing affairs would have been unseemly and calculated to defeat the purpose in view. Nothing in the published report of the National Council of Women Committee has induced us to alter this opinion. The questionnaire sent out was, as we have already pointed out, inquisitorial in character and in many particulars useless for purposes of comparison and information. The natural result has been that in a vast number of instances no reply was received at all, and that in others many of the queries received no answer. This is most marked in respect of salaries, under which heading the Committee wisely intimate that they "confine their remarks to the case of probationers," or, in other words, to particulars already furnished by all hospitals to the public. The number of replies received totalled 176, as compared with 514 received by the College of Nursing Committee.

---

PARTICULARS of interest elicited by the above Committee in respect of the sixty-six hospitals containing over 100 beds and the ninety-five hospitals containing under 100 beds which returned the questionnaire centre round the bedroom, bathroom, and lavatory accommodation available for members of the nursing staff. In the larger hospitals 59.3 and in the smaller 45.3 per cent. provided a separate bedroom for each nurse. The proportion of bathrooms was found to vary from one for every four nurses, to one for every twenty nurses. The proportion of w.c.s was found to vary also from one for every three or four nurses, to one for every twenty nurses. The Committee urge the provision of one bathroom for every four nurses and one w.c. for every six nurses. Without knowing how many of the sixty-six hospitals indicated above as containing over 100 beds are Poor-Law infirmaries, it is impossible to draw conclusions relating to the minute points discussed in the report. It is well-known that many Poor-Law institutions have been adapted from old buildings, and are greatly in need of re-construction. But we fear pronouncements of this vague character can have little effect. These are matters which can only be dealt with individually after skilled inspection on the spot by such an authority as the Ministry of Health having power to enforce alterations. The Committee should have discriminated between Poor-Law institutions and voluntary hospitals in preparing their statistics.

---

IN THE HOSPITAL for February 22, 1919, page 455, we gave an account of an attempt by Mrs. Fenwick and her supporters to capture the College of Nursing and appropriate one piece of work undertaken by the College through a Special Committee to consider and report upon the conditions of employment of nurses and the salaries paid to them, which report the whole nursing profession was anxiously awaiting. This unwarrantable attempt was exposed and failed, as the report of the College Council's Committee, which dealt with the returns submitted to it by no fewer than 514 institutions, shows. The actual facts and position were kept back from the House of Commons, misrepresentation being substituted with success until the true position of the training-schools and nursing opinion became known to its Members, and the Central Committee's Bill was defeated on its merits. The tactics pursued earned and won discredit. The importance of the House of Commons attempt to

**ROUND THE HOSPITALS—(*continued*).**

mislead, though morally serious, is, however, small compared with the wanton deception exhibited in the following extract from the *B.J.N.* of August 2. It runs: "We hope next week to discuss the very valuable Report issued by the Special Committee of the National Council of Women on the Economic Position of Nurses, **and in justice to the Committee publish in full the section which deals with the withdrawal of the representatives of the College of Nursing Limited.**" What will trained nurses think and properly declare concerning the statement we have printed in black type when they learn that the report of this Special Committee of the National Council contains not one word of the nearly three columns of abuse of the College of Nursing which the *B.J.N.* states it publishes in full from the report of the Special Committee of the National Council of Women? Those whom the gods wish to destroy they first drive mad. The instance here recorded is probably the most complete example of shameless moral suicide ever published.

Women who came from abroad after the outbreak of war and joined the Naval V.A.D. Nursing Service are to have repatriation overseas at the public expense, provided that they were serving in hospitals under direct naval control, on a six months' contract when the Armistice was signed. Exceptions to this rule will be considered individually. The Central Joint V.A.D. Committee are dealing with those who came over in response to an invitation from this body and were serving on November 11, 1918, in a hospital not under direct naval control. Forms of application for repatriation may be obtained from the Director-General of the Navy, 12 Charles Street, Berkeley Square, W. 1.; applications can in no case be received later than October 31.

The Royal Sanitary Institute starts the autumn session of lectures and demonstrations on Monday, September 22, for students preparing for posts as sanitary officers, women health visitors, tuberculosis visitors, school nurses and teachers, maternity and child-welfare workers, meat and food inspectors. Students have the use of an extensive Reference Library and arrangements are also made for them to borrow one book at a time from Lewis's Medical and Surgical Library on payment of a nominal fee. All information respecting terms and examinations, with a syllabus of the various courses, may be obtained from the Secretary, 90 Buckingham Palace Road, S.W. 1.

At the Health and Welfare Exhibition held in the Manchester Free Trade Hall from September 4 to 11 an American factory, the National Cash Register, Dayton, Ohio, U.S.A., was represented at a stall which attracted a large number of visitors. The factory is a model so far as regards healthful and beautiful surroundings. In addition to the ordinary activities of a welfare department, such as ambulance and rest rooms, canteens, athletic grounds, etc., the N.C.R., whilst practically making provision for everything conceivable in the way of health and comfort, has inaugurated such features as (1) sterilising of all brushes and combs used by employees daily; (2) shaving and manicuring for all connected with dining-rooms and canteen; (3) provision of baths, which may be taken in firm's time; (4) recess periods twice a day; (5) overshoes and umbrellas lent out on wet days to those employees who have come unprovided; (6) medical inspection of all new employees; (7) lifts whenever there are steep flights of stairs; (8) open-air camps hired out at moderate charges for summer holidays. The industrial betterment branch is known at Dayton as "the house of usefulness." We could wish that in some of its features it could command imitation in establishments where large numbers of probationers undergo training.

Three notable contributions received by the College of Nursing towards the Nation's Fund for Nurses from the provincial centres bear witness to the continued zeal of the members. Members of the College at Hull, part of the Yorkshire Centre, have forwarded per Miss Eleanor E. Edwards, Honorary Treasurer of the Yorkshire Centre, the sum of £185, proceeds of a collection for the Endowment Fund. From the Northumberland War Hospital, Gosforth, Newcastle-on-Tyne, there has come the sum of £85 from the matron, Miss Jean P. Macleod, the proceeds of a garden party, for the Endowment Fund. Lastly, from the Derbyshire and Chesterfield Centre, through Miss Berry, North Derbyshire Hospital, comes £150 for the Endowment and Tribute Fund. The value of these contributions is enhanced by the testimony they afford of a spirit of service abroad among members all over the country towards their College.

It is very pleasant to meet in the August number of the *South African Nursing Record* an appreciative notice by Miss Alexander, Hon. Secretary of the South African Trained Nurses Association, of the annual meeting of the College of Nursing held at Manchester last June. After describing with admiration the zeal and good fellowship which characterised all the proceedings, and the splendid advances made by the College during the past year, Miss Alexander recounts some of the fertile suggestions under discussion by the members of the College and concludes with the hope that she may be able "to carry back a little of its spirit and unity and genuine enthusiasm, for the benefit of the South African T.N.A." Thus the College is already beginning to achieve one of its cherished aims, that of radiating faith and sympathy to nurses in far distant lands, bound to us by ties of kinship in one Empire, nourished on the same ideals, inspired by a common purpose.

The members of the Royal Club of Women from Beyond the Seas gathered in force at Norfolk House on September 10 to present gifts to the Duchess of Norfolk and express their appreciation of her kindness in surrendering her house for their use during

**ROUND THE HOSPITALS**—(*continued*).

the war. The Australian, Canadian, and New Zealand nursing sisters formed a semi-circle behind the Duchess, who made a telling speech in response. There is some hope that a permanent club may be secured in London for women from the Dominions who are visiting London, but Devonshire House, which was talked about in this connection, must always have been beyond the powers of the Committee, even had it not passed into other hands. Something much less ambitious would better serve the purposes of the members.

An Honours Board was unveiled in the Council Chamber of the Deptford Borough Council last week by the Mayor in honour of Sister Sophie Hilling, who died in France last October from pneumonia, at the age of 33, while on duty with the Forces. Sister Hilling was born in Deptford. She joined the Q.A.I.M.N.S.R. at the outbreak of war and was detailed to the Welsh Metropolitan Hospital at Cardiff, where she remained till she was sent abroad in December 1917. Before leaving England, the King bestowed upon her the decoration of the Royal Red Cross, 2nd Class, for devotion to duty. This is the first instance we have noted of municipal recognition of the service rendered by a nursing sister. We trust the example of Deptford may stimulate other boroughs to keep green the memory of their nursing sisters.

The enquiry which the Merthyr Guardians conducted recently into the allegations of under-feeding at their Infirmary illustrates some of the worst features of Poor-Law management. It was held in public and lasted over three hours. Without their own consent two of the nurses were subjected to a rigorous cross-examination on the subject of the alleged complaints, and although they firmly protested that they had merely grumbled among themselves and had no charges to make against the Superintendent Nurse, they were grossly persecuted with a view to obtaining admissions. Leading questions of a most unfair character were put and their replies were received with "roars of laughter." We protest strongly against the impropriety with which this so-called inquiry was carried out, and commend a perusal of the proceedings reported in the *Merthyr Express* of the 6th inst. to the attention of the Local Government Board.

The authorities of King Edward VII. Hospital, Cardiff, have revised the salaries of the nursing staff, in fulfilment of a promise made some months ago. The revised salaries for probationer nurses begin at £12 per annum and rise to £18, £24, and £35 for the second, third, and fourth years respectively. Staff nurses are to have £45 per annum, increasing by yearly increments of £5 to £60; ward sisters, £60 per annum, increasing by yearly increments of £5 to £75 per annum. In view of the financial anxiety under which at the moment the hospital is working, the decision to increase salaries does the authorities credit, and should prove a wise expenditure.

The experiments now taking place in the industrial world with a view to lessening labour and increasing output ought assuredly to find imitation in institutions. Dr. Meyer's recent report to the Industrial Fatigue Board describes the process adopted at the Derwent Iron Factory, Derby, in order to cut out useless work. As early as 1915, by the consent of the employees, a careful study, with aid of a stop-watch, was made of all the movements required in each operation. The tools and materials were arranged in a standardised manner and a standard set of movements with a standard time was adopted for each process. Some such system introduced into the training of probationers could facilitate to an undreamed of extent the work of every ward. Every keen observer must have been struck with the prodigious waste of domestic energy fostered by bad systems in every household. In institutions, this wastage is responsible for incessant drive and worry. It may be noticed that some attempt at this close economy of time and movement is made in every operating theatre and its extension to other departments of hospital activity would lighten the labours of every member of the staff.

The Royal Victoria Infirmary, Newcastle-upon-Tyne, has long been in great straits for want of better accommodation for the nursing staff. Even before the war began, the committee had under consideration the enlargement of the Nurses' Home in connection with other building extensions. For the last five years, all deliberations have been suspended; but we learn from the recent report of the infirmary that the special committee appointed for this purpose has been revived and that plans for additions to the Nurses' Home are being got out. Meantime, the pressure on space having become acute, the house committee have purchased Cross House, Upper Claremont, to accommodate some of the nurses. Twenty-one nurses left for military or naval work during the past year. It is noteworthy that two sisters have lately retired from the nursing staff after thirty years devoted work, Sister Whitfield and Sister Nichol.

One of the nursing sisters in the employ of the Birmingham Education Committee lost her life on September 8 in a collision which took place between a tramcar and the motor-omnibus used for conveying cripple children to the special school. At the time when the omnibus was struck by the tramcar it contained two nurses and one little boy. Sister Hampton, aged thirty-two, was pitched out into the road and sustained a severe fracture of the skull. She was taken at once to the General Hospital, but life was found to be extinct. The other nurse, Miss Gordon, escaped with a cut on her face and some severe bruises, and the child was also bruised and frightened.

